# Research Progress on the Geomechanical Properties of Block-in-Matrix Rocks

**DOI:** 10.3390/ma17051167

**Published:** 2024-03-01

**Authors:** Songfeng Guo, Qianhui Wei, Shengwen Qi, Lei Xue, Bowen Zheng, Hongjian Wang, Jinxuan Li, Shuaihua Song, Ning Liang, Yu Zou, Zhiquan Huang

**Affiliations:** 1 Key Laboratory of Shale Gas and Geoengineeering, Institute of Geology and Geophysics, Chinese Academy of Sciences, Beijing 100029, China; z20211020254@stu.ncwu.edu.cn (Q.W.); qishengwen@mail.iggcas.ac.cn (S.Q.); xuelei@mail.iggcas.ac.cn (L.X.); zhengbowen@mail.iggcas.ac.cn (B.Z.); lijinxuan0703@163.com (J.L.); shuaihuasong@mail.iggcas.ac.cn (S.S.); liangning@mail.iggcas.ac.cn (N.L.); zouyu@mail.iggcas.ac.cn (Y.Z.); 2 Institutions of Earth Science, Chinese Academy of Sciences, Beijing 100029, China; 3 University of Chinese Academy of Sciences, Beijing 100049, China; 4 College of Geosciences and Engineering, North China University of Water Resources and Electric Power, Zhengzhou 450046, China; wanghj@ncwu.edu.cn (H.W.); huangzhiquan_183@126.com (Z.H.); 5 School of Civil Engineering, Chang’an University, Xi’an 710061, China; 6 Luoyang Institute of Science and Technology, Luoyang 471023, China

**Keywords:** Bimrock, engineering geomechanics, rock mass strength, rock mass classification, failure modes

## Abstract

The differences in geomechanical properties and the uncertainty in the spatial distribution of Bimrock pose significant challenges to the construction and disaster prediction of geotechnical engineering. To clarify the geomechanical characteristics of Bimrock, this paper summarizes the basic concepts and classification methods of Bimrock at home and abroad. It discusses the methods and characteristics of determining the geometric features of Bimrock blocks and explores the influencing factors and laws of failure modes and strength under different stress states of Bimrock. The study finds that the failure mode of Bimrock is mainly influenced by factors such as block proportion, degree of welding between blocks and matrix, strength ratio between blocks and matrix, and geometric properties of blocks. Among these factors, block proportion is the most significant, and the degree of welding is a controlling factor. However, due to the complexity of Bimrock structures, there is a lack of applicable methods and mechanical models for the evaluation of geomechanical characteristics of Bimrock in engineering practice. This article also explores the influence and research methods of the geological characteristics of Bimrock in slope and tunnel engineering and, finally, provides prospects for the future research trends relating to Bimrock.

## 1. Introduction

Bimrock (mélange), initially defined as a rock mass with internal continuity that can be mapped at a scale of 1:24,000 or smaller, consists of rock fragments of different sizes, whether external or original, embedded in a matrix typically formed through shear processes such as fault rocks, volcanic mudstones, glacial deposits, and weathered rocks [1]. From an engineering perspective, it can be simplified as a block-in-matrix structure, referred to as Bimrock [2].

Bimrock has been discovered in over 60 countries [2], with the most in-depth studies conducted on the Franciscan mélange, covering approximately one-third of Northern California [3,4]. Other notable examples include the following: the Ahauser dam breccia in the northern part of the Rheno–Hercynian zone of the Variscan Mountains in Germany [5,6]; the Shale–Limestone Chaotic Complex found in the Santa Barbara open-pit mine in Italy [5,6,7,8,9,10,11]; and the Sille Agglomerate discovered in Koimesis Tes Panagias Church and Sekili hill settlement carved in Turkey [12,13]. China also has extensive occurrences of Bimrock, particularly in several large Bimrock belts on the Tibetan plateau, including the Luhuo–Daofu ophiolite Bimrock belt [14], the Ganzi–Litang accretionary Bimrock belt [15], the Jiali–Palong Zangbu ophiolite Bimrock belt [16], the Jinsha River accretionary complex Bimrock belt [16], the Bangong Lake–Nujiang subduction accretionary complex Bimrock belt [17], the Lancang River accretionary complex Bimrock belt [18], and the Yarlung Zangbo River ophiolite Bimrock belt [19].

Due to the prevalence of Bimrock belts in tectonically active regions, secondary disasters such as landslides are likely to occur. The complex lithology, structure, geological hydrology, and geomechanical characteristics of Bimrock pose significant challenges to engineering projects involving underground structures, tunnels [20,21,22], and slopes [23,24,25]. Notable incidents include the Bai Ge landslide in Changdu City, Tibet, and the Temigu landslide in Batang, which dammed the Jinsha River, leading to substantial downstream losses [26,27]. Therefore, it is very important to explore the physical and mechanical properties of Bimrock.

This paper analyzes indoor and outdoor mechanical tests, physical model tests, numerical simulation methods, and conclusions from both domestic and international research. It discusses the impact of factors such as the proportion of Bimrock masses, parameters related to the number and shape of blocks, and the strength ratio of Bimrock masses to matrix on their failure modes and strength. The study aims to derive general principles and explore the impact of Bimrock on engineering projects such as slopes and tunnels.

## 2. Definition and Classification of Bimrock

### 2.1. Definition of Bimrock

Raymond [3] classified mélange and several other aliases of mélange as “block-in-matrix” structures, which is one of the numerous geological terms used to represent fragmented rocks and Bimrocks, including friction carpets, wildflysch, broken formation, argille scagliose, olistostromes, mega-breccias, sedimentary chaos, mixed clays, etc., as listed by Laznicka [28] and Neuendorf et al. [29]. However, in engineering applications, the focus is more on the mechanical properties and structure of the block, and detailed classification based on rock evolution is not necessary. Therefore, Medley [2] created a neutral term “Bimrock” from Raymond’s “block-in-matrix” [3] concept. Many geological processes can produce Bimrock, including sedimentary rocks (large conglomerate blocks, slope deposits, and glacial deposits); igneous rocks (clasts in volcanic ejecta, volcanic debris, lava, and xenoliths in intrusive igneous bodies); deep-seated rocks (xenoliths in intrusive igneous bodies); brittle fractured rocks in tectonic fault zones (fault breccias); rocks formed by structural ductile deformation in fold disturbance zones (mélange); rocks formed by bottom-up processes (some sheared serpentinites); and rocks formed by chemical and mechanical weathering processes (saprolite, weathered granite, and welded conglomerate).

Formally speaking, Bimrock is a type of rock mixture composed of blocks with engineering significance accompanying a weaker matrix. The engineering significance refers to the following three points.

Mechanical specifications of blocks and matrix. The key to considering a rock mass as Bimrock is the moderately strong ratio between blocks and matrix, as shown in Table 1. This ratio can be determined based on three factors: elastic modulus, internal friction angle, and uniaxial compressive strength, as described by Medley and Zekkos [30]. For cases below the lower limit shown in the table, the failure surface is more likely to cut through rock masses rather than around them.

Specification of the maximum and minimum sizes of blocks. Medley [2] defined Lc as the characteristic engineering size, similar to a reference object in a photograph for comparing the scale of images. Lc can be (1) a scale indicator of the study area, such as $\sqrt{A}$, where A is the area of the study; (2) the maximum size of blocks $d_{\max}$ measured or estimated in the study area; (3) the thickness of the failure zone below a landslide; (4) tunnel diameter; (5) foundation width or length; (6) the size of laboratory specimens. Medley [2] defined 0.05 Lc as the lower size limit to distinguish blocks from the matrix and 0.75 Lc as the upper limit for block size, $d_{\max}$. Blocks larger than 0.75 Lc lead to a reduced proportion of matrix in local rock masses and can be considered as unfaulted rock masses composed of blocks, namely, jointed block rock masses. Blocks smaller than 0.05 Lc have negligible effects on the mechanical behavior of Bimrock and can be treated as part of the matrix. Therefore, at any scale, the threshold size between blocks and matrix is set as 0.05 Lc.

Specification of the volume ratio of blocks. The volume ratio of blocks should be greater than 25% and less than 75%. This criterion was proposed by Lindquist [31] and Lindquist and Goodman [32]: When the volume fraction of blocks is less than 25%, the overall mechanical behavior of the rock is similar to that of the matrix; if the proportion of blocks in the matrix exceeds 25%, the overall strength of the matrix will be enhanced at the characteristic engineering size of rock laboratory tests. When the volume ratio exceeds approximately 75%, the impact of the matrix on strength can be negligible, and it can be regarded as a jointed block rock mass.

However, recent research results indicate that, even if the strength contrast is not satisfied (e.g., Kahraman and Alber [5,6]; Kahraman et al. [33,34]) or the proportion of blocks does not fall between 25% and 75% (e.g., Delenne [35]; Afifipour and Moarefvand [36,37,38]), as long as there is a heterogeneous structure of matrix-embedded blocks, it can be defined as Bimrock, and its research methods and mechanical principles are still applicable.

### 2.2. Classification of Bimrock

The classification of Bimrock is currently based on the following three main methods: classification based on internal heterogeneity of blocks and the degree of disruption [39]; classification based on genesis [40]; and classification based on the degree of welding between blocks and matrix [41].

**Figure 1:**
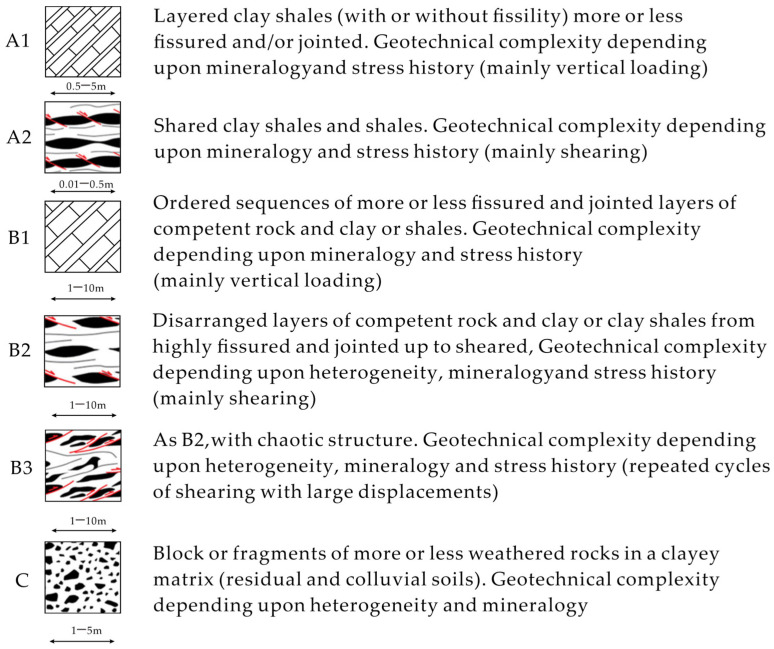
Classification of complex strata (modified from [56] (The red line shows the shearing action).

Esu [39] subdivided complex formations into three main groups (Figure 1), each with different internal heterogeneity and degrees of disruption. The first group (“A” group) includes coherent sedimentary (rock) units, ranging from well-layered and well-bedded sediments to sheared sediments. The second group (“B” group) includes sedimentary (rock) units with varying degrees of disruption, ranging from fractures in an orderly sediment (i.e., well layered; subgroup “B1”) to chaotic rock units with blocked clasts (subgroup “B3”), where the blocks are embedded in a softer and sheared matrix. The last group (“C” group) includes highly heterogeneous sedimentary units consisting of weathered rock fragments embedded in clay matrix (e.g., residual soil and colluvium). The labels “residual soil and colluvium” for the “C” group indicate that these blocks are formed by weathering of parent rock and surface gravity transport (colluvium, landslides, etc.).

Festa [40] believes that the bim structure depends largely on the formation process and classifies mélanges into sedimentary mélanges, tectonic mélanges, and bottom-up mélanges [40,42,43,44]. Under the influence of crustal movements such as earthquakes, underground fluids entrain magma and erupt to the surface, forming bottom-up Bimrock [45]. Tectonic mélanges are mainly formed by shear processes and are characterized by an “ordered structure”, i.e., scale-independent repetition, typically with elongated blocky bodies that have a lens-like or uniformly distributed aspect ratio. Sedimentary mélanges typically form in submarine debris flows, namely, landslides [46,47,48]. In contrast to tectonic mélanges, sedimentary mélanges have a highly disordered arrangement and different block shapes randomly distributed in a finer-grained matrix [49,50].

**Figure 2:**
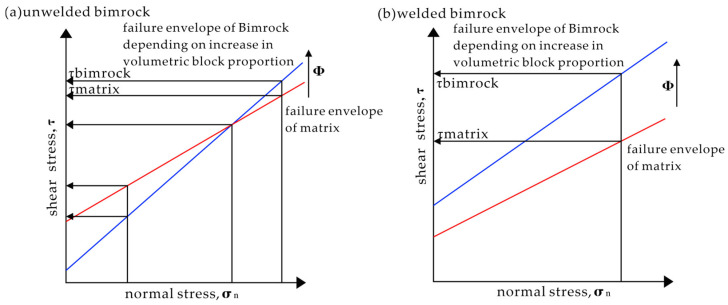
Schematic explanation of the mechanical behavior of a welded and unwelded Bimrock (modified from [54]).

Altinsoy [41] adopted the Bimrock classification method proposed by Riedmüller et al. [51], categorizing Bimrock into welded Bimrock and unwelded Bimrock. For the former, the boundary strength between the matrix and blocks is considered approximately equal to the strength of the matrix, while, for the latter, the contact strength between the matrix and blocks is less than the strength of the matrix. Volcanic Bimrock formed under high-temperature and high-pressure conditions often belongs to welded Bimrock [52,53]. Figure 2 schematically illustrates the geomechanical characteristics of welded and unwelded Bimrock [54]. For unwelded Bimrock, the internal friction angle is greater than that of the matrix, and the cohesion is less than that of the matrix. Therefore, with increasing principal stress, the shear stress of Bimrock gradually changes from being less than that of the pure matrix to being greater than that of the pure matrix. The failure envelope of the mélange rocks changes with the increase in block proportion. For welded Bimrock, both cohesion and internal friction angle are greater than those of the pure matrix, so, under the same principal stress, the shear stress of Bimrock is always greater than that of the matrix. For unwelded Bimrock, the blocks can be easily separated from the matrix, but it is difficult to obtain undisturbed rock mass. For welded Bimrock, it is almost impossible to separate the block from the matrix without damaging the rock mass, but undisturbed samples can be obtained by means such as drilling and coring [54].

Based on these three simple classification methods of Bimrock, further refinements can be made. Nikolaidis and Saroglou [55] proposed a method based on six parameters (linear volume ratio of block bodies, strength of Bimrock, complexity of the matrix, complexity of Bimrock, classification of block bodies, and direction of block bodies) to characterize rock masses with bim structures, which can be directly assessed in the field. Napoli [56] considered rock type (water sensitivity), degree of anisotropy, internal disruption of complex formations, and block volume ratio (VBP) and proposed a more comprehensive classification method that allows for detailed classification based on laboratory mechanical tests and on-site photos.

## 3. Research on the Measurement Method and Uncertainty of Geometric Features of Blocks in Bimrock

Due to the unique structure of Bimrock with matrix-enclosed blocks, the content and shape parameters of block play a significant role in the mechanical properties of Bimrock. The effective determination of block content is crucial and can be expressed as volumetric block proportion (VBP), rock block proportion (RBP), or equivalent block proportion (EBP) [57]. Based on stereometric principles [58], the following four measurement methods can be used to determine the block content of Bimrock: 0D point sampling, 1D scan lines and drilling [59], 2D photo image analysis and mapping of windows [2,5,6,11,12,13,33,34], and 3D sieving analysis [9]. According to Delesse’s principle [60], when the sample size is sufficiently large, all these measurements of different dimensions can yield the same result. Since 0D point sampling involves many uncertainties and is labor intensive, and 3D sieving analysis, while the most accurate method on a laboratory scale, is usually impossible due to the contact strength between strong block bodies and weaker matrix [61,62], the 1D and 2D methods are more widely used to determine the block volume ratio.

### 3.1. One-Dimensional Measurement

**Figure 3:**
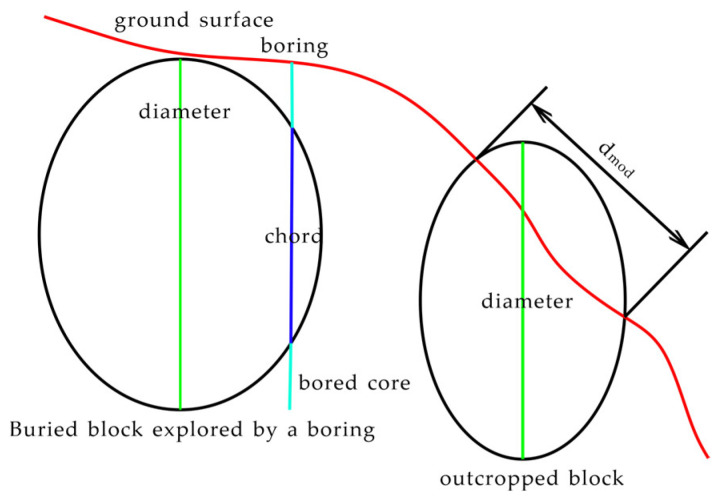
Relationship of d_mod_ or chord length to actual maximum size (modified from [30]).

The block volume ratio is related to the linear ratio of blocks, the ratio of total chord length to drilling length, or the ratio of the length of block intersected by the line traversing the exposed rock outcrop ($d_{\mathrm{mod}}$) to the total length of the outcrop [63]. When the rock is not obviously exposed and cannot be directly observed, one-dimensional measurement can be carried out by drilling and other methods, or, when the study area is large and it requires a lot of work to directly obtain the block proportion, one-dimensional measurement can be used for rough estimation first. However, estimating the three-dimensional block volume ratio using one-dimensional linear ratios introduces uncertainty because chord lengths and $d_{\mathrm{mod}}$ are generally shorter than the maximum size of block bodies (see Figure 3), and, thus, one-dimensional chord length distribution typically underestimates the actual three-dimensional block size distribution.

Although a material with strictly fractal characteristics should have a formation process that repeats itself, many scholars found that Bimrock, with a multiplicity of formative processes, also poses fractality properties. Sammis and Biegel [64] demonstrated that the size distribution of block bodies in Bimrock has self-similarity and can be described by a negative power law (fractal) distribution, where the absolute value of the negative slope of the frequency–size relationship plotted on logarithmic coordinates is called the fractal dimension (Equation (1)).

(1) $$Y=CX^{-D}$$

Here, *Y* is the cumulative block frequency for each block size, *X* is the size of the block, *C* is a constant coefficient related to the maximum block size, and *D* is the fractal dimension [62,65,66].

**Figure 4:**
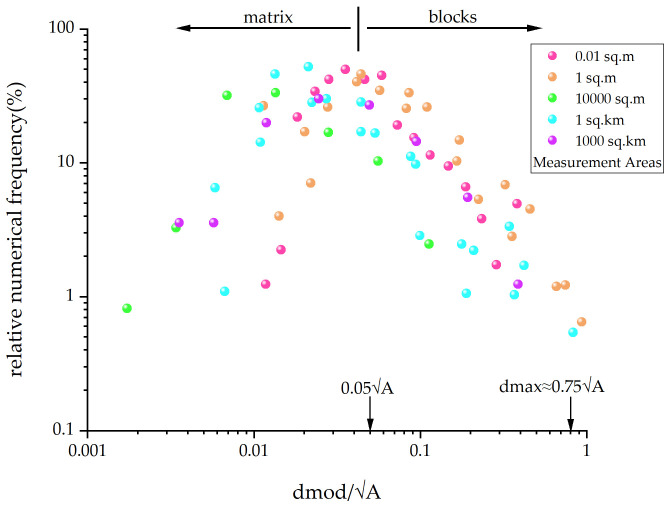
Histogram of logarithmic distribution of Franciscan mélange block size and relative frequency (modified from [30]).

Self-similarity means that, in study areas of different sizes, the size distribution of blocks has approximately the same fractal dimension [61]. Medley [2] measured $d_{\mathrm{mod}}\;$for about 1900 blocks in the Franciscan mélange, covering an area range exceeding seven orders of magnitude, as shown in Figure 4. By transforming the block size distribution at different scales to a dimensionless form using $d_{mod}$/$\sqrt{A}$, and representing block frequencies in relative terms, it was found that the fractal dimension of block size distribution measured by one-dimensional methods ranged between 1.1 and 1.7, with an overall value of approximately 1.3. It was also observed that, when $d_{mod}\;$is 0.05$\sqrt{A}$, the block frequency reaches its peak, and over 99% of block sizes are smaller than 0.75$\sqrt{A}$. Based on the fractal characteristics of the Franciscan mélange, Lindquist [31] created physical models of different block arrangement directions and simulated drilling with scan lines. The results indicated that the peak of the chord length distribution is related to the high relative frequency of the smallest size block, once again confirming that the peak of the chord length distribution can reflect the size lower limit of the block relative to the matrix. Medley [67] demonstrated through physical modeling that there is almost no equivalence between chord length distribution and actual block sizes, with a pronounced tail effect. Small chord length distribution is greater than the actual block size, but this can be corrected and transformed through estimates of uncertainty factors determined by different scan line lengths and VBP.

**Figure 5:**
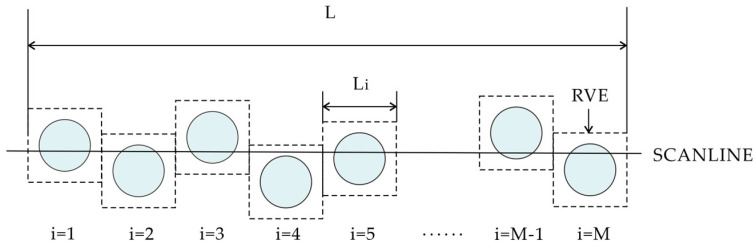
Repeated RVE array structure (modified from [70]).

However, the results of the above studies are empirical, and it is necessary to quantify the uncertainty of one-dimensional measurement estimates of three-dimensional block proportions. The magnitude of uncertainty depends primarily on the number and depth of boreholes [67], the VBP itself [68], and additional variables, including block size, shape, and distribution direction [69,70]. The introduction of the concept of the representative volume element (RVE) is the basis for studying the uncertainty of one-dimensional measurement [68,70]. As shown in Figure 5, for a given VBP, the RVE can be considered as a fixed-size circular block volume uniformly distributed in a rectangular matrix with an area ratio equal to the VBP. When a line of length L intersects a block with diameter D along one edge, n line segments of different lengths are randomly formed.

Theoretical derivation of the estimation of VBP for random variables is carried out, and the results of the scan line are extended to the expected value of three-dimensional block estimates $V_{b}$. Simulations with different lengths of scan lines show that, as L/D increases, the histogram of estimates of VBP obtained from the RVE converges to a Gaussian distribution, and convergence is better with increasing VBP. Therefore, average block size and estimated VBP can be used to quantify uncertainty. Analytical equations for the mean and variance of estimates of VBP from scan lines and normalized coefficients of variation were derived.

**Figure 6:**
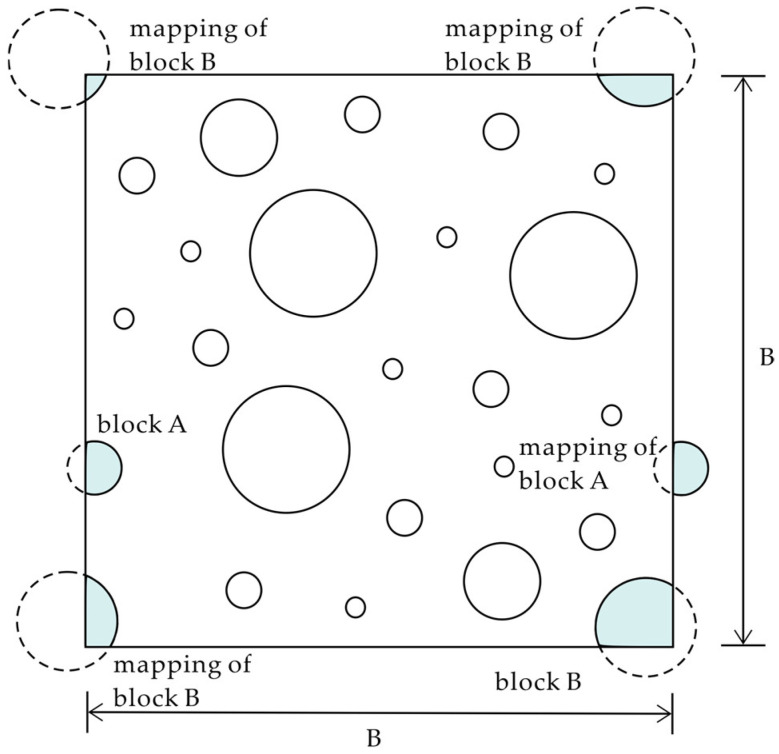
Conceptual diagram of periodic boundary (modified from [70]).

Ramos-Cañón et al. [71] reconfirmed, based on 3D numerical simulations of PLB, that the variables with the greatest impact on the uncertainty of VBP determination are the number and depth of boreholes or one-dimensional scan lines and the VBP itself; the smaller the VBP, the greater the uncertainty. Lower bounds on uncertainty estimates were also quantified. Considering the anisotropy of the actual block distribution, Tien et al. [69] proposed the concept of an RVE containing an ellipse within a parallelogram. Analytical solutions were derived to quantify uncertainty in one-dimensional scan line estimates of volume fraction for anisotropic heterogeneous materials. The method includes block aspect ratio, orientation, diameter, and volume fraction and is further simplified with intercept length. Tien et al. [72] derived an analytical solution based on the concept of the RVE to quantify uncertainty in estimating the volume fraction (Vf) in inhomogeneous materials using a two-dimensional probe, showing that the uncertainty of estimates depends on block size, measured area, and Vf level. When the RVE method is transformed into the 2D domain, it produces uneven wall (edge) effects near the edges. To reduce the impact of wall effects, Tien et al. [68] trimmed four strips from the edges of an extended square (generated domain), but this method may lead to $V_{b}$ dispersion. To address this, Lu et al. [70] adopted a periodic boundary method, as shown in Figure 6, each circle in figure represents a block. Where, if a block intersects one edge of the boundary, the external part of the block is mapped to its opposite side. This effectively avoids wall effects.

### 3.2. Two-Dimensional Measurement

Processing the two-dimensional images of Bimrock to estimate VBP requires satisfying the two following conditions to reduce uncertainty: the ratio of block length and axis being small enough and there being no obvious anisotropy. This is because, when the block distribution has obvious anisotropy, the outcrop block distribution will not reflect the internal situation of the mélange. The blocks have sufficient color contrast or clear boundaries with the matrix so that the blocks can be easily distinguished from the matrix, and the photos obtained for image processing can be more accurate in analyzing the geometric features. To determine whether there is anisotropy in the distribution of block bodies due to their random distribution, photos can be taken from multiple angles on the exposed surface of Bimrock, and the long and short axes of each block can be scanned to create a cumulative frequency distribution [52,53]. Since the distribution of blocks in Bimrock is random, it is generally considered that more than 70% of the aspect ratios of the long and short axes of blocks are less than 1.5, which can be regarded as approximately isotropic in both 2D and 3D [5,12,52].

When there is a significant contrast in color between the block and the matrix, scanning is typically performed on the circumferential surface of the core, and grayscale or RGB color photos with highlighted color tones are used for image processing [52]. Image processing methods include image classification and node counting methods. The overall goal of image classification programs is to use “supervised” or “unsupervised” classification to automatically categorize pixels into different themes [73]. Node counting methods involve overlaying a square grid on the photo and determining the percentage of each component by dividing the number of node intersections for each component by the total number of grid intersections [52]. For cores where the boundaries between the block and the matrix are not clearly defined due to severe weathering or the minerals themselves, or when the scanner accuracy cannot achieve the necessary precision due to small sizes, image processing may not automatically select blocks. In such cases, transparent film is wrapped around each core, and the boundaries are manually traced, after which the film is scanned with a scanner, and the block boundaries are redrawn for digitalization [5,12]. Digitalized images can be used not only to determine VBP but also to obtain parameters such as the number of blocks, roundness, perimeter, area, etc. [12].

According to the principles of stereovision, the uncertainty of two-dimensional measurements estimating VBP is similar to that of one-dimensional measurements and mainly comes from the sample size and the VBP itself [74]. In addition, Haneberg used Monte Carlo computer simulations to generate ellipsoids with different eccentricities, orientations, and distributions (uniform and random) to simulate rock blocks [75]. The study explored the deviation introduced when inferring 3D block distribution from 2D projections and found that the error depends largely on block shape and orientation relative to the outcrop face. Sampling of outcrop photos can overestimate or underestimate block sizes and proportions, with errors reaching up to 80%. Napoli et al. developed Matlab code to generate 3D Bimrock models with given block size distributions and different VBP values. They assessed the deviation of 2D measurement values (ABP) from VBP as a function of the study area size and adjusted ABP measurement values to estimate VBP [24].

**Figure 7:**
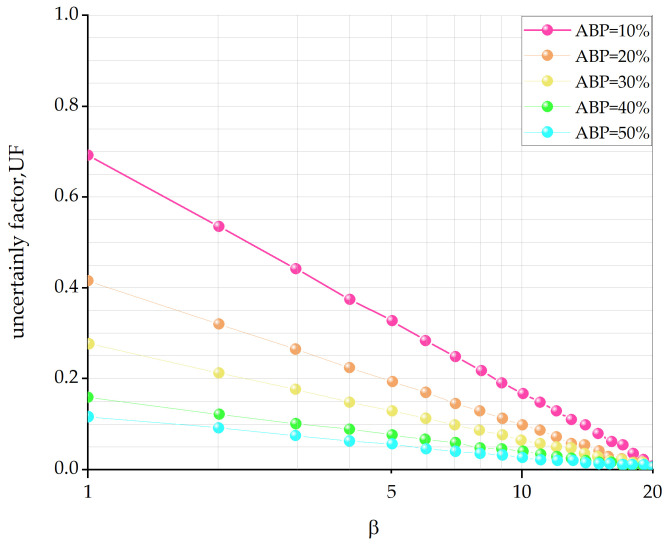
The uncertainty factor of the two-dimensional measurement VBP estimate is a linear fit on a semilog plot as a function of the two-dimensional measurement ABP (modified from [24]).

The resulting graph can be directly used or interpolated to analyze a series of ABP values (Figure 7) and sizes from outcrops, thereby obtaining estimates of VBP.

As different VBP values in Bimrock can also affect the overall density and seismic velocities, some researchers have attempted to estimate VBP values from density or velocity. If there is a significant density contrast between block bodies and the matrix, the overall density of the sample will be proportional to the VBP [31]. Kahraman et al. used regression analysis to study this and found that density is closely related to VBP and has statistical significance, making it suitable for estimating VBP [34]. However, VBP has no significant relationship with shear wave velocity and shows a weak linear relationship with compressional wave velocity. This is because acoustic wave velocities are not only related to VBP but also sensitive to block shape, size distribution, and the strength ratio of block bodies to the matrix. Mahdevari and Maarefvand conducted transverse and longitudinal wave tests on physically prepared samples and found that, for isotropic Bimrock with blocks, both transverse and longitudinal wave velocities increase with increasing VBP [76]. The estimated uncertainty depends on the maximum block size and fractal dimension. The transverse and longitudinal wave velocities decrease with increasing fractal dimension, but there is no significant trend between the variation of transverse and longitudinal wave velocities and the maximum block size, and the change in wave velocities with VBP is greater than with the fractal dimension.

## 4. Bimrock Failure Modes and Influencing Factors

### 4.1. Deformation and Failure Modes of Bimrock under Axial Compression

**Figure 8:**
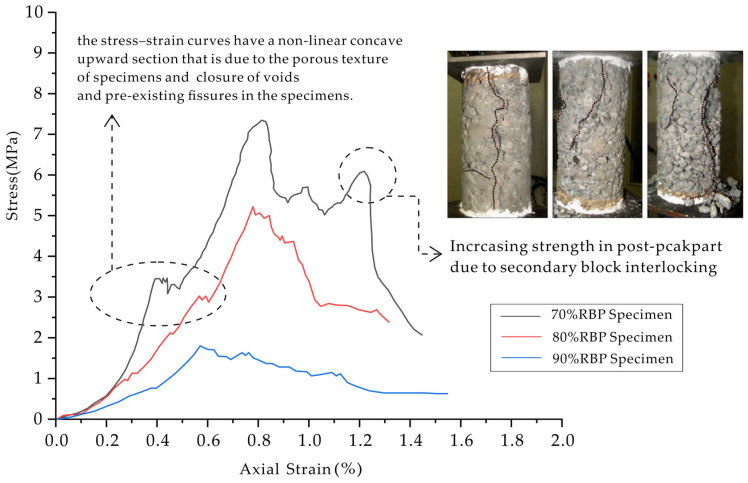
Typical uniaxial stress–strain curves of Bimrock model with different block mass ratios (RBP) (modified from [38]).

The typical failure mode of brittle homogeneous rocks in uniaxial compression tests is primarily axial splitting, where cracks propagate from top to bottom parallel or nearly parallel to the loading axis [77]. However, Bimrock, with its heterogeneous structure of matrix-embedded blocks, may exhibit interference with the original failure path. Afifipour and Moarefvand analyzed the failure process of Bimrock based on stress–strain curves (Figure 8) [38]. In the initial stage of loading, all curves show similar trends, with nonlinear convex portions in the stress–strain curves due to the specimen’s porous structure and closure of voids and pre-existing cracks. This initial nonlinearity is more pronounced in samples with a higher rock block proportion (RBP).

Subsequently, there is an approximately linear portion, followed by a nonlinear portion until the peak strength point. The initial yield strength is related to localized degradation of the bonding surface and matrix deformation, while the second yield strength is related to rock block interlocking or other interactions. Similar trends were observed in in situ shear tests conducted by Coli et al. [10] and two-dimensional numerical simulations by Xu et al. [78]. After reaching peak strength, increasing RBP in the sample transforms the deformation behavior from “rapid stress drop” to “slow stress drop with increasing strain”, and post-peak behavior depends on the block proportion. In specimens with lower RBP, the post-peak region exhibits strain softening as the dominant trend, and an instantaneous hardening phase is also observed. This unique trend may be attributed to secondary interlocking caused by block displacement. The specific failure mode is mainly influenced by VBP, block geometric properties, block–matrix strength ratio, and the degree of bonding.

The strength contrast between blocks, the matrix, and their contact points is a primary factor influencing the failure mode of Bimrock. Kahraman et al. [33] found in the study of the Misis fault breccia with uneven matrix strength that, when the matrix strength is low, the failure surface follows along the block boundaries. When the matrix strength is high or when the interblock bonding strength is higher than the strength of individual blocks under loading conditions, block fracture may occur [79]. If the strength at the contact points is low, the failure surface still bypasses the blocks. Medley and Zekkos integrated three lower bounds of block–matrix strength ratio (refer to the first section) and suggested that, if the ratio is below the lower limit, the failure surface will pass through the blocks [30].

VBP, together with the degree of bonding, often determines the failure mode. Early studies have shown [31] that, when the VBP is between 25 and 75%, for unwelded Bimrock under uniaxial compression, due to the lowest strength at the contact point, the failure surface of the mixed rock extends around the block along the contact point, and the block strength has almost no effect on the failure mode. Kahraman and Alber found that the Ahauser dam breccia, a bonded mixture, still adheres to this rule, with a VBP ranging from 28.5% to 68.3% [5]. However, when the VBP is less than 28.5%, noticeable matrix failure is observed, and, when the VBP exceeds 68.3%, the failure surface penetrates the blocks. This may still be related to the specific low block strength of the Ahauser dam breccia. For columnar specimens of Bimrock with a high block proportion, as the block proportion increases, the failure mode under uniaxial compression gradually exhibits continuous zigzag failure surfaces, multiple localized shear failure surfaces, and detachment of blocks along the edges of the specimen [36]. The way of Bimrock strength changing with VBP is determined by the bond degree between blocks. When bonding, the trend of increasing or decreasing VBP depends on the curvature degree of the failure path and whether it passes through the blocks. If the block strength is less than the matrix, the strength changing trend with increasing VBP is more pronounced.

The failure mode is also related to the overall size and fractal dimension of the blocks [80]. Due to the larger surface area of coarse blocks, when the blocks stay on the failure surface, the surface may penetrate the blocks rather than extending along the periphery of the blocks. As the VBP decreases and the fractal dimension increases, the failure morphology of Bimrock transforms from block fracture to matrix and block fracture. In addition, at a low VBP and high fractal dimension, the failure mechanism is peeling; increasing VBP and decreasing fractal dimension transition to shear failure.

**Figure 9:**
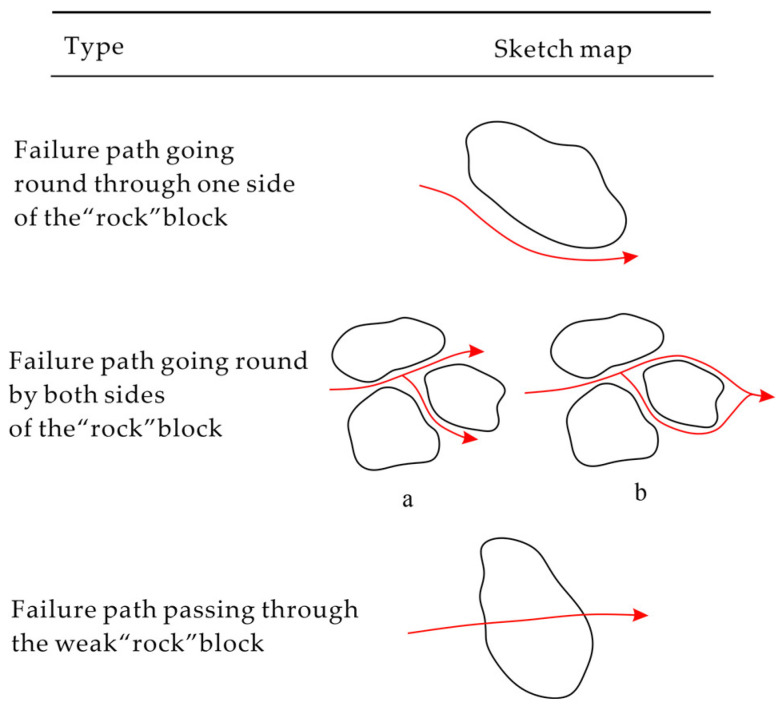
Schematic diagram of three possible failure modes of Bimrock (modified from [78]). (**a**) and (**b**) in the figure denote two failure paths going round two sides of block.

Xu et al. summarized the following failure modes under uniaxial compression of Bimrock with three schematic diagrams (see Figure 9, the red arrows indicate the destruction path) [78]:

1. Failure path around rock blocks. This type of failure occurs where the matrix is weaker than the rock blocks, and the block proportion is small, allowing enough relative space for the failure path to pass through. Examples include conglomerates with highly filled matrix, non-bonded, coarse-grained alluvial and colluvial deposits, mélanges [2,31], and agglomerates formed under high temperature and pressure conditions [52];
2. Branching along both sides of the block before merging. This situation also occurs where the matrix is weaker than the blocks but the block proportion is high and the relative positions or contacts between the blocks are small [36];
3. Penetration through rock blocks and matrix. This occurs when the mechanical contrast between the blocks and the matrix is small, or the blocks are weaker than the matrix [6].

**Figure 10:**
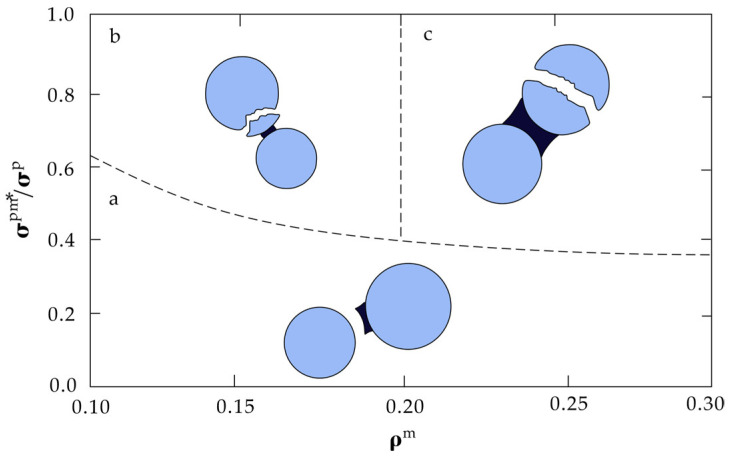
Schematic diagram of crack state changes with matrix volume fraction $\rho^{m}$ and effective particle–matrix adhesion $\sigma^{pm*}$ (modified from [35]). a denotes cracks sliding along block boundary, b denotes block surface fracturing and c denotes block fracturing extensively, which can be addressed in the main text in detail.

Considering the way the failure path passes through the block, Delenne defined the block damage value $F^{pm}$ as a function of the product of the matrix volume fraction ($\rho^{m}$) and the degree of cementation between the block and matrix ($\sigma^{pm*}$), i.e., $F^{pm}\propto \sigma^{pm*}\rho^{m}$ [35]. Figure 10 shows the expansion states of the following three kinds of cracks in the Bimrocks, $\sigma^{pm*}$, normalized by the particle tensile strength $\sigma^{p}$:

1. Below the particle damage limit *F^pm^*, cracks bypass the particles and propagate through the matrix, pores, or along the block–matrix contact surface due to high bulk content and weak cementation (Figure 10a);
2. If this limit is exceeded and $\rho^{m}<0.2$, cracks will also penetrate from the solid bridge part into the block, thus concentrating stress strongly and causing surface wear of the block (Figure 10b);
3. Above this limit and where $\rho^{m}>0.2$, cracks spread through the matrix and inside the block due to the uniform stress of the matrix, resulting in block rupture, and the proportion of damaged blocks increases with the increase in $\rho^{m}$ and $\sigma^{pm*}$ (Figure 10c).

### 4.2. Deformation and Failure Characteristics of Bimrock under Shear Action

The mechanical behavior of Bimrock under shear action is also influenced by VBP. Avşar found that the shear failure mode is more influenced by normal stress than VBP [13]. Under all normal stresses, Bimrock undergoes strain softening after reaching the shear stress peak, and specimens with higher VBP values exhibit a smaller decrease in shear stress. Under low normal stresses, shear dilation is observed regardless of VBP values, and the dilation magnitude increases with increasing VBP. As normal stress increases, vertical displacement decreases, and primarily contraction behavior is observed. Additionally, under low normal stress, both the peak and residual strengths are significantly influenced by VBP.

## 5. Analysis of Factors Influencing the Mechanical Properties of Bimrock

Due to the unique characteristics of Bimrock, its mechanical properties are influenced by various factors, including the block proportion, the orientation of anisotropic block arrangement, block quantity parameters, block shape parameters, and the block–matrix strength ratio. The main areas of influence include the uniaxial compressive strength (UCS), deviatoric stress in triaxial tests, elastic modulus (E), mechanical parameters such as internal friction angle (φ) and cohesion (c), the trend of crack propagation under different deformation and failure conditions, and the relationship between acoustic wave velocity and various influencing factors. In recent years, scholars both domestically and internationally have expanded their analysis from early single-variable studies to establishing models with multiple combined parameters [12,13,34,52,53,54,81,82,83].

### 5.1. Block Proportion

Lindquist and Goodman indicated that when the VBP is <25%, the strength of the Bimrock is approximately equal to the strength of the matrix [32]. When the VBP is >25%, the Bimrock can be considered as a joint-filled rock mass, and the strength depends on the strength of the blocks. When the VBP is between 25% and 75%, the overall strength of the Bimrock is directly related to VBP and does not depend on the strength of the blocks. Therefore, block proportion is the most significant factor affecting the mechanical properties of Bimrock. In addition, the degree of weld is a determining factor in the trend of Bimrock with varying VBP [82].

**Figure 11:**
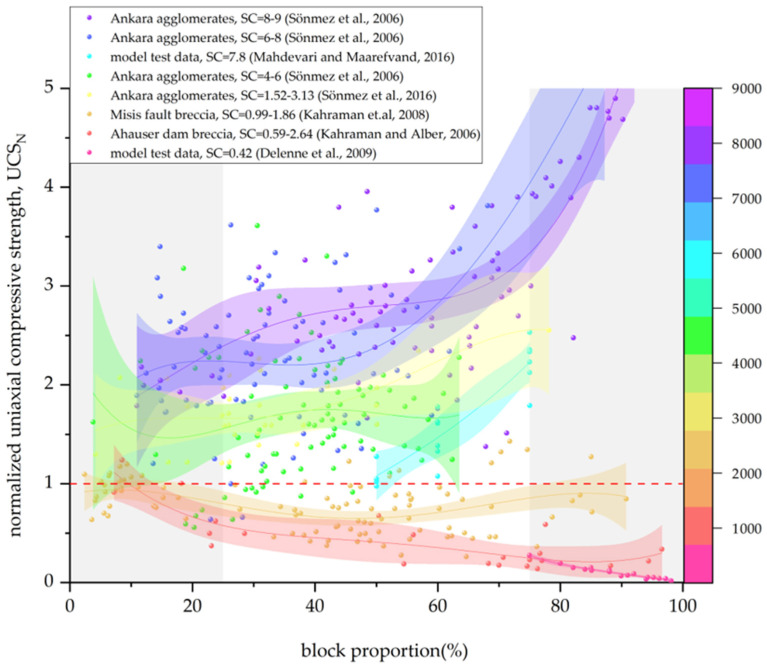
The relationship between the normalized uniaxial compressive strength and block proportion of welded Bimrock; SC is the block and matrix strength comparison. (The data in the figure are from [5,33,35,54,57,80]).

**Figure 12:**
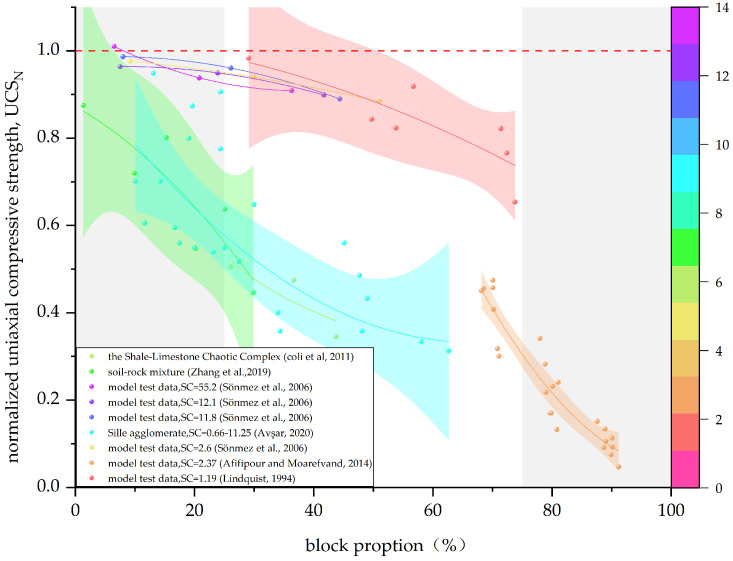
The relationship between the normalized uniaxial compressive strength and block proportion of unwelded Bimrock; SC is the block and matrix strength comparison. (The data in the figure are from [10,12,31,38,84,85]).

**Figure 13:**
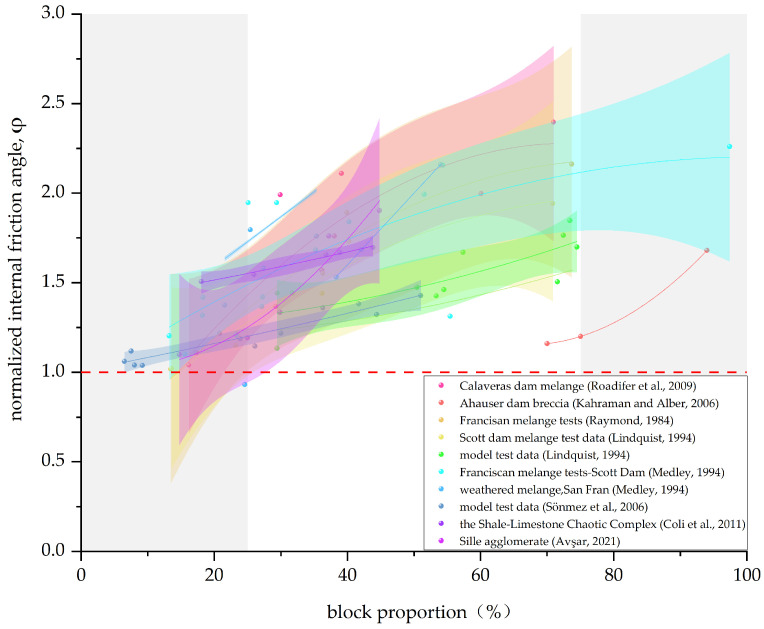
Correlation between normalized internal friction angle and block proportion. (The data in the figure are from [2,3,5,10,13,31,85,86]).

**Figure 14:**
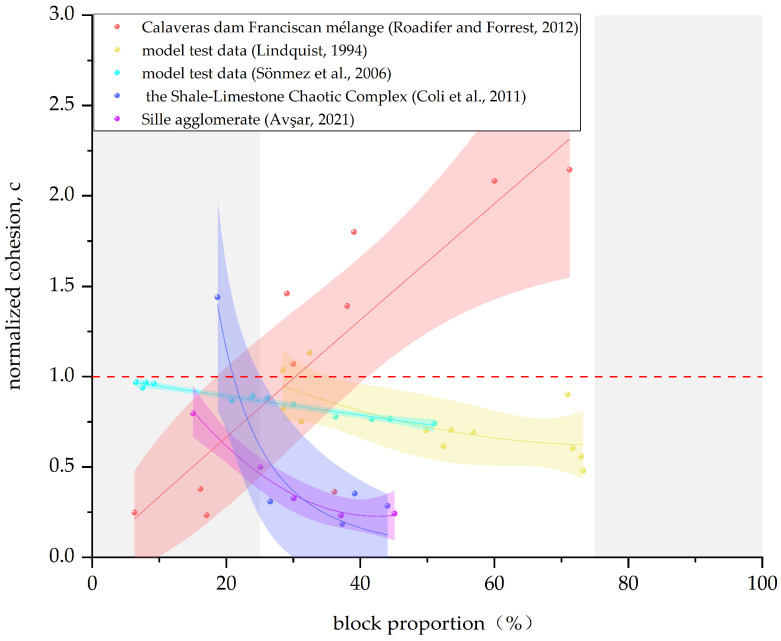
Correlation between normalized cohesion and block proportion. (The data in the figure are from [10,13,31,85,87]).

In this paper, the data given in Table 2 are summarized, and the Bimrock is classified into welded and unwelded Bimrock for the discussion of different mechanical parameters. For ease of visual statistics, Figure 11, Figure 12, Figure 13 and Figure 14 normalize the mechanical parameters shown on the vertical axis by the corresponding matrix parameters.

Figure 11 shows the trend of normalized uniaxial compressive strength (UCSN) of welded Bimrock with the change in block proportion. When the block proportion is >25%, UCSN generally increases with the increase in block proportion and is greater than 1. This indicates that the UCS of welded Bimrock is generally greater than that of pure matrix because the block strength is greater, and its presence increases the tortuosity of the failure surface, thereby increasing the overall strength of the Bimrock. Different from other welded Bimrocks, Kahraman and Alber found that the UCS of Ahauser dam breccia decreases with the increase in block proportion and is generally lower than that of the pure matrix [5]. The main reason is that the strength of the limestone matrix of Ahauser dam breccia is greater than that of the slate blocks. Similarly, for Misis fault breccia [33], there are two different trends when the VBP is below and above 25%, and there is no overall correlation, and the strength is lower than that of the pure matrix. This is because it only satisfies the bim structure, and, different from the conventional Bimrock block–matrix strength ratio, the matrix strength range is large, so there is a large difference in the overall strength of the Bimrock under the same VBP.

From Figure 11, it can also be seen that, when the block proportion is <25%, with the increase in block proportion, the overall UCS shows a downward trend or is approximately equal to the matrix strength. It can be considered that the presence of blocks destroys the heterogeneity of the matrix, causing stress concentration. Since the number is small, it is not enough to form a tortuous failure surface around the failure path. When the block proportion is >75%, UCSN still shows an increasing trend, and the increase is even greater. This is because, for samples with a high block proportion, even if the block strength is higher than the contact surface, the failure path is forced to pass through the blocks [57]. Delenne [35] found through model experiments that the uniaxial compressive strength under high block proportion has a good linear relationship with the product of the matrix volume fraction (ρm) and the block–matrix bond degree (σpm*). However, contrary to the trend of Sönmez et al. [57], this is because the LECA beads selected by Delenne [35] as the model experiment have a block strength smaller than the matrix.

Figure 12 shows the trend of normalized uniaxial compressive strength of unwelded Bimrock with the change in block proportion. In contrast to the trend in Figure 11, the strength is generally lower than that of the pure matrix, indicating that blocks have a weakening effect on the overall strength. This is because the contact surface between the blocks and the matrix is the weakest part of unwelded Bimrock, and the presence of blocks increases the contact surface area, making the failure surface easier to expand. Avşar observed that the failure surface of core samples mainly passes through the boundary between blocks and matrix, verifying that the boundary strength of Sille Agglomerate is the lowest, belonging to unwelded Bimrock, and UCS is significantly negatively correlated with VBP [12]. Therefore, changing the roughness and size distribution characteristics of the boundary length is crucial for predicting the UCS of unwelded Bimrock. The data from Afifipour and Moarefvand show that the strength of unwelded Bimrock with a high block proportion decreases more significantly with the increase in block proportion [36]. Medley proposed scale independence related to VBP [2].

Kahraman and Alber explained that the scale effect weakens with the increase in VBP [5]. Afifipour and Moarefvand also observed that, for Bimrock with a high block proportion, UCS values are independent of specimen size, and the difference between VBP and RBP is very small [36]. In addition, unwelded Bimrock shows the same decreasing trend regardless of the block proportion. This is because, at a high block proportion, unwelded Bimrock can be regarded as an extremely fragmented jointed rock mass [31]; therefore, the strength still decreases.

Block proportion also affects the shear properties of Bimrock. Figure 13 and Figure 14 show the change in normalized internal friction angle and cohesion of Bimrock with block proportion, respectively. From Figure 13, it can be seen that, whether it is welded or unwelded Bimrock, the internal friction angle increases with the increase in block proportion because the block proportion changes the tortuosity of the failure surface of the Bimrock and is not affected by the strength of the contact surface [13,31].

Coli et al. [10] demonstrated through unconventional in situ direct shear tests that the internal friction angle has a good linear positive correlation with VBP (R^2^ = 0.85); Avşar [13] also proved a similar linear relationship through direct shear tests, with a high correlation coefficient of 0.912. Early research [31] believed that, for unwelded Bimrock, when the block proportion is relatively small (0–10%), there is almost no increase in the internal friction angle relative to the matrix; when the block proportion is greater than 75%, the internal friction angle reaches the rest angle, and it can be assumed that there is no or very low interlocking contact between the blocks, and there is almost no increase with the increase in VBP [88]. However, in the studies of Medley [61], Coli et al. [10], and Avşar [13], it is shown that, when the block proportion is less than 30%, the internal friction angle also changes significantly. The data on Ahauser dam breccia from Kahraman and Alber [6] also show that the internal friction angle of Bimrock still changes significantly with VBP under a high block proportion (>75%). By comparing the effects of block proportion on uniaxial compressive strength and shear strength of unwelded Bimrock, it can be considered that the internal friction angle depends only on the matrix itself and the block proportion. For uniaxial compressive strength, the block direction and roundness also have a significant effect. Kalender et al. have given a good predictive model for this relationship [82].

Figure 14 shows that the cohesion changes with VBP are similar to the normalized uniaxial compressive strength. Welded Bimrock shows a positive correlation, and unwelded Bimrock shows a negative correlation. Also, the cohesion of welded Bimrock is greater than that of the pure matrix, while the opposite is true for unwelded Bimrock. Roadifer and Forrest [87] obtained a linear relationship between the cohesion of welded Bimrock and block proportion under two matrix strengths, indicating that the cohesion of welded Bimrock under weaker sheared shale matrix is smaller than that under a stronger matrix, and there seems to be a 25% threshold to distinguish between the two amplitude changes of cohesion. For unwelded Bimrock, the increase in the contact surface area between blocks and the matrix weakens the overall degree of welding, and Avşar [13] obtained a good inverse proportional linear relationship between cohesion and VBP (R^2^ = 0.857) through direct shear tests. In the study of Coli et al. [10], cohesion suddenly decreases at a VBP around 20–25%, indicating that, when this threshold is reached, blocks begin to control the overall bonding strength of Bimrock.

**Figure 15:**
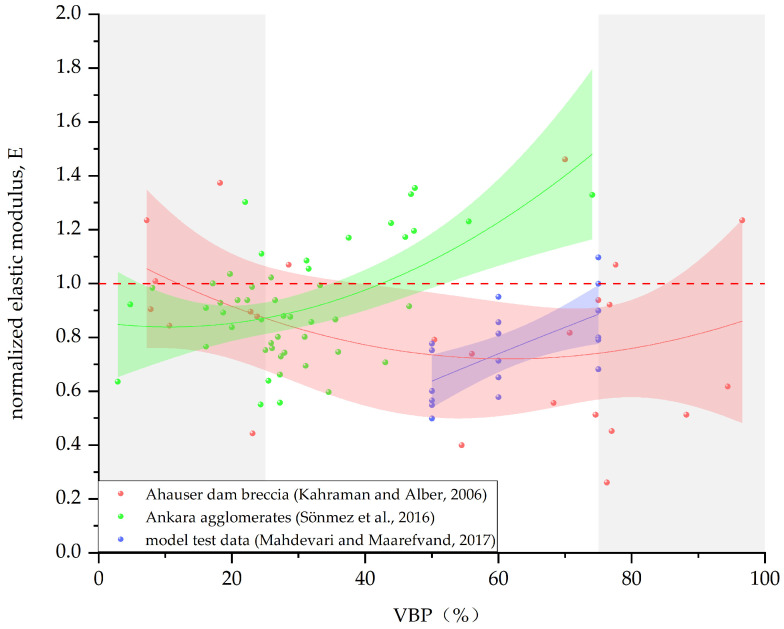
Relationship between the normalized elastic modulus E and the block proportion. (The data in the figure are from [5,54,76]).

From the influence of block proportion on the stress–strain curve of Bimrock, it can be seen that block proportion also affects the elastic modulus E of Bimrock. Since the blocks reduce the uniformity of stress transfer, the elastic modulus of Bimrock is usually smaller than that of the pure matrix. Figure 15 reflects the trend of elastic modulus with VBP. According to the test results of Kahraman and Alber [5], there is no obvious relationship between E and VBP, and it only has a good correlation when the VBP is between 20% and 70%, and other data points in other regions are more discrete. From the data of Sönmez et al. [54], it can be seen that the elastic modulus is weakly positively correlated with VBP. If introducing the block number Bc and the line roughness fractal dimension *D*, a combined parameter $CP=\frac{B_{c}(1-(\frac{VBP}{100}))}{100(D-1)},$ it is found that elastic modulus and CP have a good correlation. Mahdevari and Maarefvand [76] found through reshaping specimens that, with the increase in VBP and the decrease in fractal dimension, the deformation modulus of Bimrock increases. The high correlation factor multivariate regression analysis shows that there is an indirect relationship between the deformation modulus and the fractal dimension and a direct relationship with the maximum block size and VBP.

### 5.2. Block Quantity Parameters

Block quantity parameters include the number of blocks contained in a single specimen (Number of Counted Blocks, denoted as BN, or Block Count, denoted as BC), and the block size fractal dimension (Fragmentation Fractal Dimension, denoted as DF or D).

The block size fractal dimension represents how many blocks of different sizes are present, with the formula shown in Equation (1). The block size fractal dimension of Bimrock is generally obtained using two-dimensional image processing methods. Its 3D fractal dimension is equal to the 2D fractal dimension plus one, usually between 2 and 3 [89], and sometimes exceeds 3 [90]. When the fractal dimension is greater than 3, Bimrock is mainly composed of fragmented blocks. If the fractal dimension is less than 3, larger blocks dominate. If the fractal dimension equals 3, blocks of all sizes have the same proportion, and the distribution function is based on the total weight’s uniform distribution function [80].

**Figure 16:**
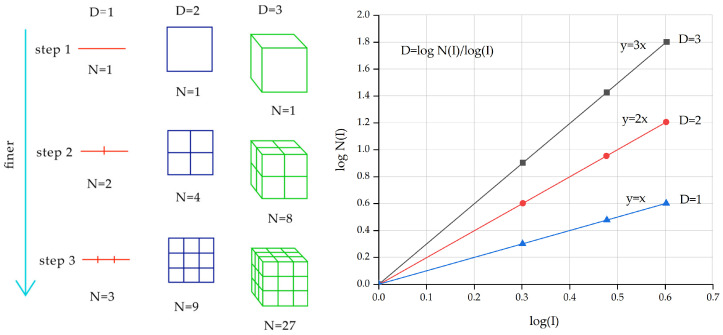
Determination of fractal dimensions in line (1D), square (2D), and cube (3D) (modified from [54]).

The calculation process of the fractal dimensions (1D, 2D, and 3D) for the same shape is shown in Figure 16 [54]. Here, it is necessary to distinguish from the roughness fractal dimension (DR), which reflects the irregularity of the blocks.

**Figure 17:**
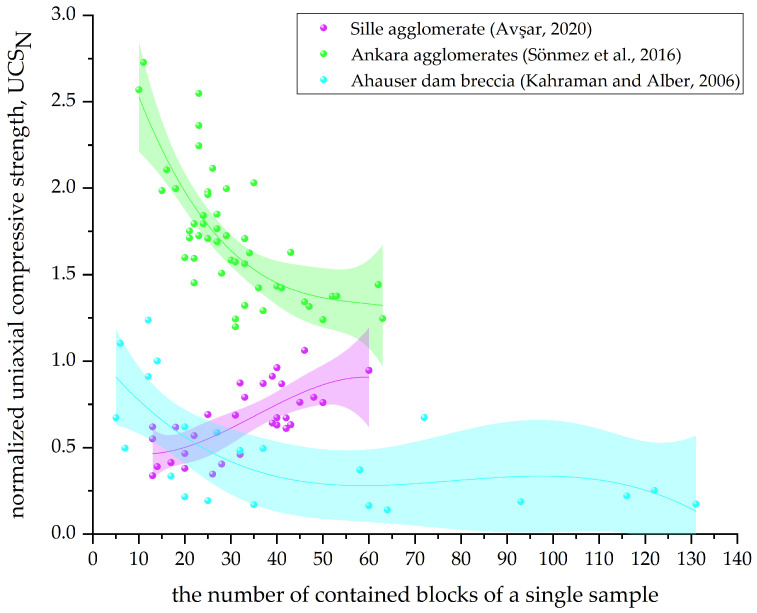
Relationship between the normalized uniaxial compressive strength and the number of contained blocks of a single sample. (The data in the figure are from [5,12,54]).

Block quantity parameters are usually obtained using two-dimensional image processing methods. These two parameters are often considered in conjunction with VBP because, under the same VBP, a larger DF implies more fragmented blocks and hence a larger BN [12]. Under the same DF, a larger BN implies a larger VBP. From the data of Kahraman and Alber [5] (Figure 17), it can be seen that the more blocks there are, the smaller the UCS. This is because the block strength of Ahauser dam breccia is smaller than that of the matrix, and the possibility of the failure path passing through the blocks increases with more blocks. However, for Ankara agglomerate [54], its block strength is higher than that of the matrix, and UCS still shows a negative correlation with BN. The original paper [54] explains that BN increases the weakest structure of the block–matrix contact surface. However, Ankara agglomerate is a typical welded Bimrock, and, as shown in Figure 11, UCS increases with the increase in VBP. This also indicates that the contact surface strength is not the weakest. Therefore, this paper believes that the complexity of the failure mode of Bimrock needs to be considered. It can be considered that, under the same VBP and VBP < 75%, as BN increases, there are more fragmented blocks, and the failure path no longer follows the edge of the blocks but directly passes through the matrix. The impact of contact strength gradually decreases, so UCS decreases.

**Figure 18:**
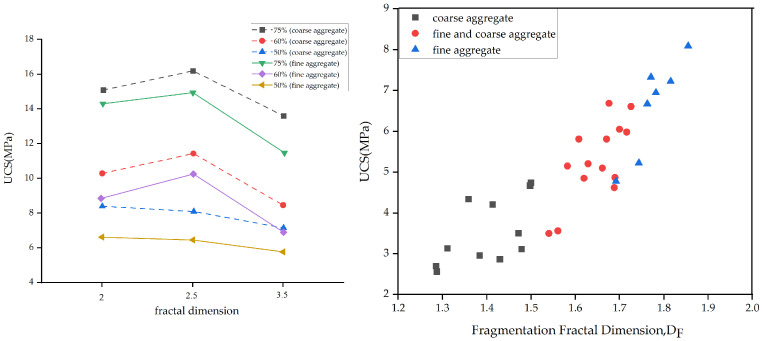
The relationship between the compressive strength of physical samples and the fractal dimension and the bulk thickness (modified from [12,80]).

From the data of Mahdevari and Maarefvand [80], it can be seen that, when the block proportion is relatively high, and the fractal dimension increases from 2 to 2.5, UCS slightly increases. This is because, when the fractal dimension is small, the gaps between large blocks are filled with matrix and air. When the fractal dimension is high, small blocks can fill the gaps between large blocks, making the overall structure denser, thus increasing strength. Avşar’s data [12] also reflect this pattern (Figure 18). However, when the fractal dimension increases from 2.5 to 3.5, the strength decreases. This is because, with the continuous increase in small blocks, shear failure has not yet spread to the interior of the specimen, and there is an overall transition from shear failure to peeling off along the surface of the specimen.

### 5.3. Block–Matrix Strength Ratio

From Figure 11, it can be observed that, for welded Bimrock, there seems to be a trend where the greater the strength contrast (SC) between blocks and matrix, the larger the normalized strength of Bimrock. However, on the one hand, it is necessary to consider that, when the SC is small, the failure surface is more likely to pass through the blocks. When the SC is large, the failure path becomes more tortuous. Both scenarios can increase the overall strength. Therefore, for different Bimrock types, it is necessary to find the threshold of the SC or welding degree to determine the trend of the effect of SC on overall strength. As for non-welded Bimrock (Figure 12), the failure surface will generally bypass the blocks so SC has no effect on its strength [57].

## 6. Impact of Bimrock on Geological Engineering

Due to the uncertainty and variability of Bimrock, its engineering behavior is more complex than other rock-soil materials. It is necessary to consider not only block quantity and size parameters and block–matrix strength ratio, but also the arrangement of blocks relative to engineering features such as slope inclination and tunnel excavation direction, which can have significant effects on engineering projects.

### 6.1. Slope Engineering

Khorasani et al. first introduced the term “bimslope”, defined as a Bimrock slope with matrix-embedded blocks [91]. Due to the difficulty in conducting in situ tests on Bimrock slopes, indoor model tests and numerical simulations are often used for analysis. Khorasani et al. [92,93] established an indoor physical model and conducted tests using a tilting table apparatus, measuring the face angle threshold of a bimslope during failure. Medley and Sanz [94] and Minuto and Morandi [95] developed a simple two-dimensional model simulating blocks with a horizontal rectangle, stating that a three-dimensional model is needed for Monte-Carlo-type random simulations to better approximate real-world conditions. Montoya-Araque et al. [96] and Napoli et al. [23,25] utilized the finite element method (FEM) and Limit Equilibrium Method (LEM) to simulate the uncertainty of actual block distributions, emphasizing the crucial role of random methods in Bimrock simulations.

**Figure 19:**
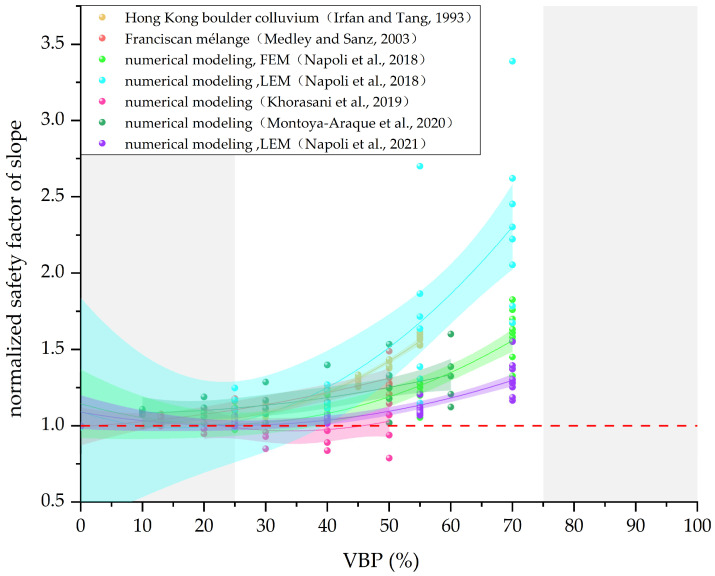
Relationship between normalized safety factor of slope and VBP. (The data in the figure are from [22,23,84,93,94,96]).

The impact of VBP (Volume Block Proportion) on the safety factor of Bimrock slopes remains significant. Figure 19 illustrates the trend of the VBP’s influence on the safety factor in various studies, with the safety factor normalized by dividing it by the safety factor of pure matrix. When block sizes and arrangements are randomly distributed, the presence of blocks increases the complexity of the failure surface, leading to an increase in the internal friction angle, regardless of the degree of welding. Khorasani et al. [92,93] found that the internal friction angle has a greater impact on the safety factor than cohesion in Bimrock slope stability. Therefore, the safety factor of Bimrock slopes is generally positively correlated with VBP, surpassing that of slopes composed solely of pure matrix [95]. Additionally, as the VBP increases, especially exceeding 20%, the arrangement of blocks becomes more complex, resulting in increased variability in the safety factor and greater uncertainty in slope stability analysis [92,93].

The degree of inclination of blocks relative to the slope angle plays a crucial role in the safety factor. If the block shapes exhibit anisotropy, and the angle between the block and the slope is relatively small (<15°), the blocks may not significantly affect slope stability [97]. Simultaneously, due to the minimal strength of the block–matrix contact surface, it might even reduce the slope safety factor [25,94]. As the angle between the block and slope increases, the impact of blocks on the safety factor also becomes more significant. Typically, when rock blocks are inclined at 0° relative to the horizontal plane, they tend to generate the most convoluted and irregular failure surface, resulting in the highest safety factor, particularly when the VBP exceeds 40% [25,97].

The degree of welding, represented by the strength of the block–matrix contact surface, also affects slope stability. To further investigate the impact of contact strength parameters, Khorasani et al. [97] defined the Interface Strength Ratio (*ISR*) with Equation (2).

(2) $$ISR=\frac{C_{1}}{C_{M}}\cdot \frac{\tan\left(\varphi_{1}\right)}{\tan\left(\varphi_{M}\right)}$$

where *C* is cohesion, *φ* is the friction angle, and *I* (interface) and *M* (matrix) respectively refer to the contact surface and the matrix. The value of *ISR* varies between 0 and 1; for welded Bimrock, the *ISR* value is 1. Khorasani et al. demonstrated through the establishment of a finite element model that, under unchanged conditions, reducing *ISR* significantly decreases the stability of the bimslope [97]. Additionally, the influence of the friction angle between the contact surface and the matrix is more significant than cohesion [92,93].

In the areas where the failure surface of Bimrock slopes crosses at the engineering scale, the blocks are often unevenly distributed, leading to the presence of lean block zones and rich block zones [98]. This results in different regions of the same slope having varying internal friction angles, altering the failure path. Therefore, when modeling, the consideration of Monte Carlo random generation of blocks for the entire slope needs to account for actual working conditions.

### 6.2. Tunnel Engineering

The heterogeneity of Bimrock also poses significant challenges to tunnel engineering. One of the main construction issues is working under mixed face conditions that involve materials with different excavation characteristics. The pronounced spatial variations in rock mass stiffness and strength reduce the predictability, adversely affecting the project [20,99], especially when the block size approaches the excavation scale. Therefore, when tunneling in Bimrock formations, it is necessary to anticipate some complex scenarios such as: the necessity of using different excavation methods (such as drilling and blasting) in the same round; frequent stress and deformation variations in both rock mass and support, where the heterogeneity of structural Bimrock leads to stress concentration in blocks, potentially resulting in sudden brittle failure [100,101]; possibility of various failure mechanisms, including excessive over-excavation, face and crown collapse, brittle failure of hard blocks, and long-term creep of the matrix; and non-uniform distribution of groundwater, potentially leading to significant water influx, especially in fault zones [102].

On the other hand, the matrix during tunnel excavation may undergo significant, possibly time-dependent deformations, with the dominant rock mass behavior outside the excavation area influencing the response. Isolated blocks within the excavation area may contribute to the stability of the tunnel face but only temporarily affect the overall stability of the tunnel. Assessment of displacement monitoring data, such as displacement vector maps, time histories, deflections, and trend lines [103,104,105,106], indicates that displacement magnitudes generally increase with a decrease in the block volume ratio.

The position of relatively rigid blocks relative to the excavation perimeter plays a crucial role in the surrounding rock mass behavior [21]. Ignoring the presence of blocks can lead to severe overestimation of displacements and the extension and shape of plastic zones, with shear stress, displacement, and plastic zones strongly influenced by nearby blocks and their dimensions. Even when the VBP is equal to 25%, the presence of blocks may result in significant changes in rock mass strength. With an increase in VBP, this overestimation phenomenon becomes more pronounced [22]. However, lower VBP values (<25%) produce the opposite effect, attributed to block-deficient zones near the tunnel causing stress and shear strain concentration, thereby reducing rock mass stability.

## 7. Conclusions and Outlook

The unique structure of Bimrock, characterized by the inclusion of blocks within the matrix, along with significant differences in strength between the matrix and blocks, uneven distribution of blocks, and the anisotropy of blocks and matrix, has a profound impact on engineering construction and the prediction of induced disasters. Investigating the structure and strength characteristics of Bimrock and understanding the similarities and differences in various engineering applications are of great engineering and theoretical significance. Among these, the block volume percentage (VBP) is the most crucial factor influencing strength, but separating blocks from the matrix is often impractical. Internationally, one-dimensional scan lines and two-dimensional digital image processing methods are primarily used to obtain the VBP. However, the non-uniformity and anisotropy of block distribution in Bimrock, as well as the scale effect caused by small sample sizes, can introduce biases in VBP predictions. Therefore, corrections based on specific geological conditions are particularly important. In terms of strength analysis, since Bimrock is not a single rock mass and obtaining intact rock samples is challenging, strength determinations are typically based on uniaxial and triaxial compressive strength tests and shear strength tests on artificial Bimrock or a small number of intact samples. Predictive models are constructed or numerical simulations are performed to extend the findings to practical applications.

In specific engineering projects, slope engineering primarily considers the relative positions of block orientations to the slope surface and the distribution of blocks, while tunnel engineering focuses on variations in block distribution along the excavation direction and their relative proportions to the matrix. Both aspects can be used for short-term predictions of mechanical behavior through the development of numerical models.

Based on the analysis in this paper, it is believed that, although some research has been conducted on Bimrock, there is currently no available method or model for evaluating the engineering geological and mechanical properties of Bimrock for practical applications. Future research should address the following aspects.

Currently, predictive models for Bimrock primarily consider factors such as VBP, block shape, number, and block-to-matrix-strength ratio. The quantification of bonding degree is not taken into account in these models. The quantification of bonding degree is mostly limited to the establishment of numerical models for slopes. Therefore, the influence of welding degree should be studied.

The impact of roundness or block roughness on overall strength has been studied to some extent. Simultaneously, consideration should be given to the influence of block shape parameters on failure paths, expanding the scope to predict failure surfaces in engineering.

Presently, research on mechanical properties is confined to static responses in laboratory mechanical experiments. However, in real working conditions, dynamic loading conditions such as earthquakes, leading to stress disturbances, need to be considered.

Currently, whether in laboratory experiments or numerical simulations, the matrix is treated as a homogeneous rock mass. However, when the matrix is an anisotropic rock mass, such as laminated shale or slate, the relative positions of block arrangement and matrix anisotropy need to be considered.

When evaluating the mass quality of Bimrock, consideration should be given not only to the rock characteristics of Bimrock itself but also to the influence of structural features such as joints and faults. The feedback mechanism between the presence of blocks and the impact of structural features on rock mass quality needs further study.

## Figures and Tables

**Table 1:** The mechanics regulations of block and matrix.

Specification	Formula Expression	Reference
The ratio of block to matrix stiffness (E, Young’s modulus)	$\left(E_{block}/E_{matrix}\right)>\sim 2.0$	Lindquist [31]
The ratio of internal friction angle between block and matrix	$\left(\tan\emptyset_{weakest\;block}\right)/\left(\tan\emptyset_{matrix}\right)>1.5-2.0$	Medley [2] Lindquist and Goodman [32]
The ratio of uniaxial compressive strength (UCS) between block and matrix	${\mathrm{UCS}}_{\mathrm{block}}/{\mathrm{UCS}}_{\mathrm{matrix}}>1.5$	Medley and Zekkos [30]

**Table 2:** Data source of research on physical and mechanical properties of mélange rocks.

Name	Block	Matrix	Welded or Unwelded	References
Hong Kong Colluvium	Coarse Gravel	Soil	No	[84]
Franciscan Mélange	Limestone, Volcanic Rock, Serpentinite, Flint, and Rare Limestone and Exotic Metamorphic Rocks	Shale, Mudstone, Siltstone, Serpentinite, or Sandstone	No	[2,61,63]
Model Experiment	Sand-Silicate Cement–Fly Ash Mixture	Bentonite–Silicate Cement Mixture	No	[31]
Ankara Agglomerates	Pink and Black Andesite Blocks	Tuff	Yes	[52,53,54,57]
Model Experiment	Tuff and Andesite	Calcined Gypsum, Bentonite, Cement, and Water	No	[85]
Ahauser Dam Breccia	Shale	Recrystallized Limestone	Yes	[5,6]
Misis Fault Breccia	Chalky Limestone	Fine-Grained Red Clay Rock with Iron-Rich Clay	Yes	[33,34]
Model Experiment	Lightweight Expanded Clay Aggregate (LECA) Beads	Sealant for Building Material Joints	Yes	[35]
The Shale–Limestone Chaotic Complex	Angular Limestone	Dark Gray Clay	No	[7,8,9,10,11]
Calaveras Dam Franciscan Mélange	Gray Sandstone, Serpentinite, Siliceous Schist, Greenstone (Altered Basalt), and Blueschist (Blueschist and Hornblende Schist)	Clay Shale	No	[86,87]
Model Experiment	River Aggregates	Portland Cement and Water Mixture	No	[36,37,38]
Model Experiment	Dolomite	Portland Cement, Silica Sand, and Water Mixture	Yes	[36,37,38]
Sille Agglomerate	Andesite	Tuff	Yes	[12,13]
